# Leveraging existing opportunities for improved Orphan Drug approval in the EU

**DOI:** 10.1186/1750-1172-7-S2-A27

**Published:** 2012-11-22

**Authors:** Catarina Edfjäll

**Affiliations:** 1 Global Regulatory Affairs, Shire HGT, Eysins, 1262, Switzerland

## 

The EU regulatory framework provides opportunities for increased flexibility and speed for Orphan Medicinal Product (OMP) marketing authorisations. Nevertheless the yearly rate of OMP approvals is not increasing [1] and available tools and procedures are used infrequently.

According to a review of approval timelines from 2001-2010 [2], FDA median total review time for OMPs (235 days) was almost 5 months faster than the median EMA review time (381 days). Furthermore, FDA approves OMPs 87 days faster than novel therapies overall, whereas EMA takes 15 days longer. One driver for this striking difference between FDA and EMA review time for OMPs seems to be FDAs granting of a Priority Review which was the case for 78% of orphan product applications between 2006 and 2010 [3]. An Accelerated Review at EMA was granted only for ~3% of approved OMPs since 2001 [4]. Conditional Marketing Authorisation (CMA) is another tool that could help shorten time to availability of new OMPs, unfortunately only ~5% of approved OMPs have benefited so far [4].

Early dialogue and agreement between the applicant and EMA scientific bodies on required data would allow more timely alignment and predictability, thus supporting increased use of Accelerated Review and CMA. Coupled with more flexibility on study design for OMPs, e.g., greater acceptance of surrogate endpoints, single well-controlled trials, and data packages supplemented with post-marketing and compassionate-use data, this would create a more favourable environment for OMP approvals in the EU.

Other approaches for speedier OMP approval should be explored, including a 'rolling application' that would allow initiating review of parts of the dossier prior to validating the entire application. Additionally, increased collaboration between EMA's scientific bodies could help streamline the various regulatory procedures between orphan designation and approval, e.g. parallel discussions with the Paediatric Committee, the Committee for Advanced Therapies and the Scientific Advise Working Party. The success of such measures will require a close dialogue between the applicant and EMA. Applicants for any procedures regarding orphan drugs would benefit from a continuous support from EMA’s orphan drug sector as they have an in depth understanding of the special challenges OMP developers face.

Taking an OMP through all regulatory hurdles until approval in a more timely manner requires increased regulatory flexibility and predictability. Although regulatory procedures in the EU are working well, improvement could be achieved by increasing the use of existing tools and more creative thinking on conditions for approval of OMPs in the EU.
